# News and Notes

**Published:** 1908-10

**Authors:** 


### NEWS AND NOTES

---

Rr. J. Cheston King has just returned from the Fenwick Sanatorium, Abbeville, La.

---

Dr. J. H. McDuffie, of Columbus, Ga., made a short visit to Atlanta during September.

---

Dr. Henry R. Slack, of LaGrange, was in Atlanta during September.

---

Montreal will hold during the first two weeks in November

Dr. Leonard Smith, of Edgewood, Ga., is out again after his recent severe illness.

---

Dr. L. C. Fisher spent a portion of September in New York and other eastern cities.

---

Dr. Al Fowler is at his office regularly again after an extended tour of Europe.

---

Dr. W. L. Champion is home again after a protracted stay in Europe.

---

a tuberculosis exhibition for educating the people of that city in the most recent methods of combating this deasese.

---

The American Dermatological Association held its annual meeting in Annapolis and Baltimore, Md., September 24-26.

---

Dr. Willis Jones has been appointed attending surgeon to Grady Hospital. Succeeding Dr. W. P. Nicholson, who recently resigned. Dr. Nicholson's resignation marks the close of twelve years continuous and useful service to this hospital.

---

The approaching wedding of Dr. Ed. Crawford, of Atlanta, and Miss Gray, of College Park, on October 28th, is a social event of much interest locally, and to Dr. Crawford's many friends in the profession.

---

Dr. Thomas Hall, of Macon, passed through Atlanta on his way home from New York. Dr. Hall has been away from his practice for some time, further equipping himself for his specialty, eyes, nose and throat.

The meeting of the Seventh Congregessional Association of Medicine at Cartersville on the fourteenth of this month was both pleasant and instructive. Some very interesting papers were scheduled. Dr. McRae, of Atlanta, and Dr. T. D. Coleman, of Augusta, President of the Georgia State Medical Association, were among the expected visitors.

---

The International Congress on Tuberculosis at the recent meeting in Washington, D. C., placed itself on record against Dr. Robert Koch's views that bovine tuberculosis rarely causes human tuberculosis. The following resolution, which was unanimously addopted, will tend to settle this question for a while at least:

"Resolved, That the utmost efforts should be continued in the struggle against tuberculosis to prevent the conveyance from man to man of tuberculosis infection as the most important source of the disease.

"That preventive measures be continued against bovine tuberculosis, and that the possibility of the propagation of this to man be recognized."

---

### HYPODERMIC TABLETS FOR USE IN DISEASES OF CHILDREN.

*Editor*:

Early in my pediartic practice I encountered a difficulty in connection with making from standard adult-dose hypodermic tablets, the modifications in dosage appropriate to infants and young children. One has either to divide the tablet up in a sort of hit or miss fashion or dissolve it in water and calculate the number of drops containing approximately the desired dose.

Either method is clumsy, inexact and furthermore, in the latter instance, takes the time that is more needed in the emergency of the case.

To obviate these difficulties, I have had Mess. Sharpe & Dohme to make a list of tablets for hypodermic medication in which the dose of the drug or drugs has been made suitable to infants and young children.

I feel confident that this innovation will appeal ot the profession and that they will find the tablets useful. There are few physicians, especially those concerned in the treatment of di-ease in children who have not at one time or another been hampered by the lack of such a convenience in their practice. The following is the present list of tablets in common use:

Morphine sulphate gr. 1-48, atropine sulphate gr. 1-1200.

Strychnine nitrate or sulphate gr. 1-360.

Atropine sulphate gr. 1-800.

Nitroglycerine gr. 1-600.

Nitroglycerine gr. 1-600, strychnine sulphate gr. 1-200.

In addition to the foregoing I have requeested the manufacturers to make a tablet composed of morphine sulphate gr. 1-25 with atropine sulphate gr. 1-300. These can be divided in half, giving the dose usual in cholera infantum in an infant one year of age, that is: morphine sulphate gr. 1-50, atropine sulphate gr. 1-600.

SAMUEL A. VISANSKA, Ph.G., M. D.,
318-319-320 Prudential Building.

---

### FULTON COUNTY MEDICAL SOCIETY.

CARNEGIE LIBRARY, AUGUST 6, 1908.

REPORTED BY R. R. DALY, M. D.

DR. STIRLING PRESIDING.

*Dr. Archibald Smith* read a paper upon "Obstetrical Complications." He described at length the course of pregnancy and the parturition of a negro with a very small pelvis, whom he had advised to submit to Caesarian Section. Forceps were used, the child's head injured somewhat and the mother's perinaeum torn and her right leg partly paralyzed. He believed that though she went through labor contrary to his expectation, she and the child would have been better off and submitted to less risk if the operation had been permitted.

*Dr. Strickler* agreed that Caesarian section should have been performed or premature labor induced because of the small pelvis. Also he suggested syphysiotomy because naturally there is often some separation at the pelvis arch. He reported in one case separating the pelvis successfully with the forceps.

*Dr. Oglesby* said that a doctor was warranted in not continuing with a patient who refused his advice as this patient did.

*Dr. Todd* spoke in support of the paper complimenting Dr. Smith upon his skill and fidelity to his patient under adverse circumstances.

*Dr. Smith* in closing said he could not agree that a doctor should not treat a case such as his because the patient would not follow his advice in every feature.

*Dr. T. Gillard* presented a paper "A General Consideration of the Epithelia Found in Urine and Their Differentiation as an to Correct Diagnosis."

*Dr. Willis Jones* disagreed with the essayist and said that no one could tell where the epithelium came from with sufficient definiteness.

*Dr Strickler* said he had recently tried it without success.

*Dr. Block* said the microscope is only an aid and that the clinical symptoms must be considered in connection with its findings. He agreed with Dr. Jones as to its incertitude.

Dr. Todd said that the paper illustrates one of the dangers of specialism. Many advances are made, or seem to be made, but we can't accept them all or depend absolutely upon them without danger.

*Dr. Gaillard* replying, referred to many German investigators who claim to make the differentiation all along the uriniferrous tract and prove them later by the clinical symptoms.

---

### REGULAR MEETING FULTON COUNTY MEDICAL SOCIETY, AUGUST 20, 1908.

---

*Dr. P. Calhoun* exhibited a case of frontal sinus trouble due to a blow in March, 1907. Cerebral symptoms followed and partially subsided but severe pain continued. A nasal polyp gave a clue to the trouble and transillumination showed a shadow in the right minence. The X-ray showed a large sinue extending past the middle line. He operated May 26, 1908, and tried to establish drainage through the nose, but was unsucecssful. External drainage was made and the sinus filled with healthy granulations nicely. Only a small scar remains and practically no deformity. Headaches all disappeared and patient is comfortable in every way.

*Dr. Calhoun* also exhibited a case of rapidly growing carcinoma in the tongue of a negro.

*Dr. Claude Smith* gave his paper on the "Diagnosis of Diptheria."

*Dr. Daly* in discussion complimented Dr. Smith upon the good work the health board is doing along this line and noted the convenience with which culture tubes could be secured. He suggested that the throat applicators should be made of some flexible material so that smears could be obtained from the naso-pharynx and the larynx which can not be done with the straight swab.

*Dr. P. Calhoun* spoke of the cause and treatment of diptheria and noted that negroes do not have it probably because they are better nose breathers than the whites.

He said that conjunctional smears made before cataract operations often showed pseudo-diptheritic germs that were of the same family as the Klebs-Loeffler bacilli.

*Dr. Block* said he had cases of persistent bacilli several months after the patient seemed well. One child had five attacks three months apart which he believed were due really to the one infection never completely removed.

*Dr. Willis Jones* asked about the curative effect of antitoxin.

*Dr. Oglesby* asked what was the proper time for injections.

*Dr Armstrong* stated that the third day was the latest date at which antitoxin was affective and added that deaths came after the 14th day.

*Dr. Boynton* said that recent antitoxin seemed weaker than the older ones. formerly 4,000 units used to be enough, now 12,000 to 24,000 are needed. He thinks we are inclined to wait too long for the second dose. The pain after each injection is pitiful, yet they should be repeated after 8 hours.

*Dr. L. B. Clark* said he never saw any harm from antitoxin. He uses it at once and gave 20,000 recently with good results.

He added that we don't keep children quiet long enough. They get out to play and infect other children before their throats are clean.

*Dr. Duncan* spoke of the importance of the clinical symptoms and said the case was diptheria whether the cultures showed bacilli or not, if the clinical picture was such as to indicate it. Antitoxin has never done any good in his hands.

*Dr. Block* said the formation of the membranes is not directly due or necessarily dependent upon the bacilli, and that careless smearing is not efficient to find the germ. This explains many of the slips in diagnosis by the culture tube. He would rely upon bacterial signs more than upon the clinical, but if the later were present, he would not wait 24 hours for the culture before giving the antitoxin.

*Dr. Stirling* commended Dr. Smith's paper and agreed with Dr. Clark upon the importance of continued rest for the child.

*Dr. Smith* closed by asking that smears be carefully taken beneath the membrane if possible. Cases in which no germs appeared in this way were not diptheria even if there were membrances an prostration. He hesitates to give antitoxin as a prophylactic because there have been sudden deaths from its use in this way. He advises the use of antitoxin when case is profoundly intoxicated even before laboratory report.

*Dr. Block* read his paper upon "Exopthalmic Goitre." Discussed by Dr. LeConte.

---

### REGULAR MEETING FULTON COUNTY MEDICAL SOCIETY, SEPTEMBER 3, 1908.

*Dr. Hoke* reported a case of sacro-illiac dislocation illustrated with skiagrams. Several other diagnosis had been made, but the shadow finally showed the trouble clearly. The right ilium was pushed backward out of place. The literature on this subject is scarce and practically nothing is said about method of reduction.

The femur was used as a level to force the ilium into place while the pelvis was fixed and patient lying on left side. Plaster of Paris cast applied over hip and half way up the chest. Pain of five years duration was promptly relieved.

*Dr. Hoke* reported a case of toxic arthritis of the finger joints in which there were spindle shaded fingers due to deposits in the joints. The points were opened, fibrous tissue and some bits of bone removed with excellent results. Skiagrams illustrated the conditions before and after operation.

He used cocaine, but would prefer a general anaesthetic in another similar case.

*Dr. C. R. Andrews* read his paper upon "The Treatment of Liaauses by Bismuth-Vaseline Injection."

Dr. Lockey asked as to its value in frontal sinuses.

*Dr. Goldsmith* related three cases, one a fistula in the sacrom and the third with no bone involvement in the axillary region all of which were greatly benefited by this injection.

*Dr. Roy* asked for indication for second injection.

*Dr. Hoke* spoke of the simplicity of the procedure.

*Dr. Andrews* replied saying that Beck reports a case of cure of antrium disease. There is no good reason why it should not be used in any sinus. The second injection may be given at the end of a week and repeated as often as is necessary. The bismuth not only forms trabeculae upon which granulations may be supported, but it is rendered radio active by the X-ray and stimulates healing. Vaseline should be used instead of albolene.

*Dr. L. M. Gaines* read a paper on "The Practical Value of Blood Examinations."

*Dr. Paulin* discussing said the blood examination taken alone was of less value than many supposed. He told of a case of typhoid running ordinary course. Then temperature went to 107.2, Lencocytes 6000, Polymorpho-nurclear Lencocytes 59-60% Perforation was suspected. No operation was done however. Chills recurred in 4 days. It developed patient has hernia and staphylo coccus aureus caused chill.

In two other cases the lencocyte count showed perforation before the clinical picture indicated it.

He agrees with essayist in his conclusions as to lenkoemias and penucious anaemias where the examination is most valuable. He related three cases of leukoemia mistaken for pregnancy in which blood examination cleared diagnosis. The haeumolytic property of the blood in carcinomas is attracting attention and he looks for developments along that line.

Dr. Claud Smith agreeing with the essayist, mentioned the facial color of the cases as often being confusing. A rosy hue sometimes takes the place of the yellow of jaundice. An examination may show anemia here.

*Dr. Armstrong* told of operation upon a case of gangrenous appendix because the blood indicated it while the general symptoms did not.

*Dr. Gaines* replied and closed saying that constant practice was necessary to complete the technique. He laid stress upon the value of the differential count.

*Dr. Claude Smith* addressed the society in the interest of an ordinance to go before the City Council in relation to preventing the propagation of flies. All refuses, manures, etc., are to be properly cared for. The Society gave its indorsement to the measure.

---

### REGULAR MEETING FULTON COUNTY MEDICAL SOCIETY, SEPTEMBER 17, 1908.

*Dr. Huguley* exhibited a thyroid gland removed a week ago. The goitre was of the exopthalamic type and displayed many toxic symptoms. These disappeared with the removal of the gland.

*Dr. Harris* gave an exhaustive paper on "Sprue."

*Dr. Todd* said he has seen cases of sprue without recognizing them because the investigations made by Dr. Harris had not then been given to the profession.

*Dr. J. L. Campbell* agreed with the essayist in the value of early diagnosis of this disease and related some distressing cases he had seen.

*Dr. Harris* in closing said that there was much pellagra in Georgia and that it closely resembled sprue at first. All such cases should receive special attention in the reasonable hope preventing the disease and eventually of getting rid of it.

*Dr. Roy* read on the subject "Exploratory Puncture of the Membrane in Certain Diseases of the Middle Ear.

*Dr. P. Calhoun* could not fully agree that puncture should be made when for exploratory purposes only. He wanted more indications than Dr. Roy had presented. He feared turning a case of catarrhal trouble into a furulent one.

*Dr. Wheeler* agreed with Dr. Roy as to the usefulness of the puncture.

*Dr. Daly* agreed with Dr. Roy and added that the membrane should be incised much more frequently than it is in cases of children who were in great pain and who would not permit of other measures of relief. The pain disappeared and there was much less likelihood of pus than if the secretions were left to make their own opening.

*Dr. Stirling* complimented Dr. Roy upon his paper and agreed with him essentially.

*Dr. Roy* in closing said that he never had much difficulty after paracentesis and he thought the laity ought to be instructed that puncture of the drum membrane did *not* mean deafness as many of them suppose.

